# Research Progress of Non-Noble Metal Catalysts for Carbon Dioxide Methanation

**DOI:** 10.3390/molecules29020374

**Published:** 2024-01-11

**Authors:** Yingchao Cui, Shunyu He, Jun Yang, Ruxing Gao, Kehao Hu, Xixi Chen, Lujing Xu, Chao Deng, Congji Lin, Shuai Peng, Chundong Zhang

**Affiliations:** 1 School of Energy Science and Engineering, Nanjing Tech University, Nanjing 211816, China; cuiyingchao@njtech.edu.cn (Y.C.); 202361108022@njtech.edu.cn (S.H.); lincj@njtech.edu.cn (C.L.); 202121008109@njtech.edu.cn (S.P.); 2 State Key Laboratory of Materials-Oriented Chemical Engineering, College of Chemical Engineering, Nanjing Tech University, Nanjing 211816, China; 202121104510@njtech.edu.cn (J.Y.); 202161104182@njtech.edu.cn (K.H.); c.xx@njtech.edu.cn (X.C.); xulujing@njtech.edu.cn (L.X.); dengc@njtech.edu.cn (C.D.)

**Keywords:** CO_2_ hydrogenation, methanation, thermocatalysis, non-noble metal catalysts, mechanism

## Abstract

The extensive utilization of fossil fuels has led to a rapid increase in atmospheric CO_2_ concentration, resulting in various environmental issues. To reduce reliance on fossil fuels and mitigate CO_2_ emissions, it is important to explore alternative methods of utilizing CO_2_ and H_2_ as raw materials to obtain high-value-added chemicals or fuels. One such method is CO_2_ methanation, which converts CO_2_ and H_2_ into methane (CH_4_), a valuable fuel and raw material for other chemicals. However, CO_2_ methanation faces challenges in terms of kinetics and thermodynamics. The reaction rate, CO_2_ conversion, and CH_4_ yield need to be improved to make the process more efficient. To overcome these challenges, the development of suitable catalysts is essential. Non-noble metal catalysts have gained significant attention due to their high catalytic activity and relatively low cost. In this paper, the thermodynamics and kinetics of the CO_2_ methanation reaction are discussed. The focus is primarily on reviewing Ni-based, Co-based, and other commonly used catalysts such as Fe-based. The effects of catalyst supports, preparation methods, and promoters on the catalytic performance of the methanation reaction are highlighted. Additionally, the paper summarizes the impact of reaction conditions such as temperature, pressure, space velocity, and H_2_/CO_2_ ratio on the catalyst performance. The mechanism of CO_2_ methanation is also summarized to provide a comprehensive understanding of the process. The objective of this paper is to deepen the understanding of non-noble metal catalysts in CO_2_ methanation reactions and provide insights for improving catalyst performance. By addressing the limitations of CO_2_ methanation and exploring the factors influencing catalyst effectiveness, researchers can develop more efficient and cost-effective catalysts for this reaction.

## 1. Introduction

Fossil fuels have been extensively used since the Industrial Revolution, which has not only promoted social progress but also brought about global energy shortages and environmental pollution [1]. The massive emission of greenhouse gases, particularly CO_2_, has continuously raised the global average temperature, resulting in the rise of sea levels, the melting of glaciers, and the frequent occurrence of extreme climate events in recent years [2]. Controlling carbon emissions is an important strategic measure for the international community and governments to deal with these problems [3,4]. However, fossil fuels still account for the main body of the energy structure, and it is difficult to achieve effective control of CO_2_ in the short term [5]. At the same time, CO_2_ is an important C1 resource, which can be captured and reacted with hydrogen to realize resource utilization [6], which can not only reduce carbon emissions but also obtain high-value-added chemical products [7,8], and carbon capture and storage (CCS) is also largely mature at the technical level [9].

CO_2_ conversion pathways include photocatalysis, electrocatalysis, and thermal catalysis [10]. Thermal catalysis requires a relatively low reaction temperature, produces few by-products, and has a high conversion rate [11]. In recent years, with the development and utilization of renewable energy, hydrogen can be obtained from electrolytic water, wind energy, solar energy, etc. [12]. Hydrogen is stable and renewable; its energy is high; and it can react with CO_2_ to achieve a low-carbon cycle [13]. The reaction of CO_2_ with hydrogen can produce formic acid, methane, methanol, dimethyl ether, and its derivatives [14,15]. The methanation process of CO_2_ is simple, and the reaction conditions are mild [16]. Methane can be used as fuel or a raw material for other chemicals [17,18]. Moreover, the methane generated can be directly transported by the existing natural gas pipeline network [19]. Therefore, it has high research value in realizing the large-scale application of CO_2_ hydrogenation in industry.

CO_2_ methanation is an exothermic reaction, and it is more favorable to the reaction at low temperatures, but the dynamics limit its industrial application [20]. It was originally used to remove trace amounts of CO and CO_2_ from H_2_-rich feed gas to prevent catalytic poisoning [21]. With the emergence of the oil crisis, CO_2_ methanation was expected to generate methane as a substitute for natural gas, but due to the low reaction rate, low CO_2_ conversion, and low CH_4_ selectivity, CO_2_ methanation has not been used on a large scale in the industry [22]. In recent years, CO_2_ methanation has been extensively studied, and various scholars have explored the mechanism, kinetics, and thermodynamics of the reaction and studied the design of the reactor to improve the reaction efficiency [23]. Finding suitable catalysts to increase the rate of the CO_2_ methanation reaction and improve CO_2_ conversion and CH_4_ selectivity is the focus of research [24].

At present, the most studied catalysts for CO_2_ methanation are noble metal catalysts and non-noble metal catalysts. Noble metal catalysts mainly include Ru, Rh, Pt, Pd, Au, etc. [25]. Although noble metal catalysts show high activity, they are generally expensive, which is not suitable for large-scale use in industry [26]. Non-noble metal catalysts are mainly Ni, Co, Fe, Mo, etc. [27]. The Ni-based catalyst has been widely studied for its high methanation activity, strong hydrogen adsorption capacity, and low price [28]. However, it is prone to sintering at higher temperatures and oxidation atmospheres, so the modification of catalysts is needed to improve its catalytic performance [29]. For example, it has been found that reducing the size of Ni particles and increasing their dispersion can improve their activity [30]. In addition, the Co-based catalyst has high catalytic activity, stability, and coking resistance, but it needs appropriate reaction conditions to achieve good conversion [31]. The Fe-based catalyst has good reaction activity and a low price, but the reaction requires being carried out under high temperatures and pressures, CH_4_ selectivity needs to be improved, and it is easy to accumulate carbon [32,33]. The Mo-based catalyst has perfect vulcanization resistance and can also be used for CO_2_ methanation [34], but its activity is low in the reaction. Therefore, different catalysts have different limitations. To improve their performance in CO_2_ methanation reactions, it is generally possible to adjust the loading of active metal [35], select appropriate supports [36,37], choose the best preparation method [38], and add promoters [39,40,41]. In addition, reaction conditions such as temperature, pressure, space velocity, and the H_2_/CO_2_ ratio also affect their reaction activity [42], so appropriate reaction conditions should be selected in the research.

In this work, we review the performance of non-noble metal catalysts commonly used in CO_2_ methanation reactions based on the above content. First, the kinetic and thermodynamic properties of CO_2_ methanation are discussed. It is shown that the reaction is thermodynamically favorable at low temperatures and high pressure. From the kinetic perspective, it is found that too-low temperatures will reduce the reaction rate, so it is necessary to find catalysts that can maintain activity at low temperatures. After that, we discuss the effect of reaction conditions on catalyst activity. The appropriate reaction conditions (temperature, pressure, space velocity, and H_2_/CO_2_ ratio) are beneficial to the reaction. Then we explain and discuss the effect of support material, preparation methods, and promoters on the catalyst performance for Ni-based, Co-based, Fe-based, and Mo-based catalysts, respectively. Moreover, we also provide some ideas for the modification of the catalyst to improve the catalytic performance. Finally, we review the reaction mechanisms of CO_2_ methanation, namely the CO intermediate mechanism and the formate intermediate mechanism.

## 2. CO_2_ Methanation Thermodynamics and Kinetics

Thermodynamic analysis of chemical reactions is used to analyze whether the reaction can proceed, the chemical equilibrium state of the reaction, the thermodynamic equilibrium limit, etc. The thermodynamic reaction equation for the CO_2_ methanation reaction is shown in Equation (1) [43].

(1) $${CO}_{2}+2H_{2}\to {CH}_{4}+2H_{2}O,\;\Delta rH_{m}^{\Theta }=-165.0\;kJ\cdot mol^{-1}$$

(2) $${\Delta G=\Delta H-T\Delta S,\;\Delta }_{r}G_{m}^{\Theta }=-113.6\;kJ\cdot mol^{-1}$$

where Δ_r_H^Θ^_m_ is the standard heat of reaction and Δ_r_G^Θ^_m_ is the standard Gibbs free energy difference; Equation (2) is the Gibbs free energy equation, and the Gibbs free energy difference is negative, indicating that the reaction can be carried out spontaneously, which suggests that the CO_2_ methanation reaction is feasible on a thermodynamic level.

In addition to the main reaction of Equation (1), the CO_2_ methanation process contains the following side reactions:

(3) $$CO+3H_{2}\to CH_{4}+H_{2}O,{\;\Delta H}_{298K}=-206\;kJ\cdot mol^{-1}$$

(4) $$CO_{2}+H_{2}\to CO+H_{2}O,{\;\Delta H}_{298K}=+41\;kJ\cdot mol^{-1}$$

(5) $$CO_{2}+CH_{4}\to 2CO+2H_{2},{\;\Delta H}_{298K}=+247\;kJ\cdot mol^{-1}$$

(6) $$2CO\to C+CO_{2},{\;\Delta H}_{298K}=-172\;kJ\cdot mol^{-1}$$

(7) $$CH_{4}\to C+2H_{2},{\;\Delta H}_{290K}=+75\;kJ\cdot mol^{-1}$$

(8) $$CO+H_{2}\to C+H_{2}O,{\;\Delta H}_{298K}=-131\;kJ\cdot mol^{-1}$$

(9) $$CO_{2}+2H_{2}\to C+2H_{2}O,{\;\Delta H}_{298K}=-90\;kJ\cdot mol^{-1}$$

Obviously, it is necessary to inhibit the occurrence of side reactions in order to ensure the high selectivity of CH_4_ (Equations (3)–(9)). A number of thermodynamic analyses of the CO_2_ methanation reaction have been carried out using the Gibbs minimization principle. It is shown that when the temperature is lower than 600 °C, the CO_2_ conversion rate decreases with the increase in temperature and increases with the increase in pressure. At 1 standard atmospheric pressure, when the temperature is greater than 600 °C, the CO_2_ conversion rate gradually increases with the increase in temperature, which may be attributed to the occurrence of the reverse water gas shift (RWGS) reaction, leading to the conversion of CO_2_. When the reaction temperature is 200~500 °C, the CH_4_ selectivity remains stable. However, the CH_4_ selectivity decreases with the increase in temperature when the temperature exceeds 500 °C [44]. Thus, an appropriate temperature and pressure is beneficial for the reaction to proceed. Massa et al. [45] found that the introduction of water vapor into the system slightly reduces CH_4_ yield, but it inhibits the carbon build-up to a certain extent. Baamran et al. [43] investigated the effect of different parameters such as temperature, pressure, and phenol concentration on the generation of H_2_, CO_2_, CO, CH_4_, and H_2_O. The results showed that high-temperature conditions are more favorable for the generation of H_2_ and CO, while CH_4_ is more likely to be generated at low temperatures. Finally, a series of coking reactions occur at high temperatures, which mainly lead to catalyst deactivation.

Through thermodynamic aspects, it appears that CO_2_ methanation is thermodynamically favorable at low temperatures and high pressures. However, there are multiple fundamental steps that are often catalyst dependent, and kinetic limitations are often the hurdles that must be overcome first, which requires a thorough understanding of the fundamental mechanistic steps of CO_2_ methanation to identify the key kinetic steps that can be addressed through process operation or catalyst design.

**Figure 1:**
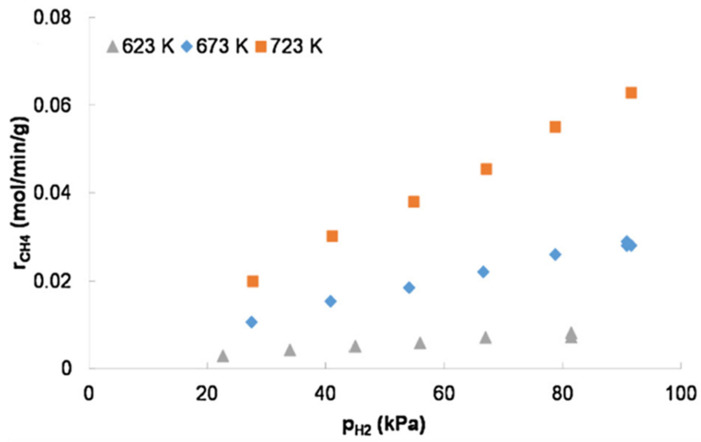
Effect of partial pressure on CO_2_ methanation on 1.5 mg Ni/Al_2_O_3_ 5 mg Ni/Al_2_O_3_ catalyst in SiC, 115 kPa total pressure and 1.06·10^6^∙h^−1^ at 623 K-130 kPa total pressure and 3.16·10^6^∙h^−1^ at 673 K and 723 K with H_2_ at 22 kPa CO_2_ partial pressure [46]. Copyright (2019) Journal of CO_2_ Utilization.

It was found that the rate of methanation of CO_2_ depends more on the H_2_ partial pressure than on the CO_2_ partial pressure at low CO_2_ conversion levels. Chanpon et al. [46] have established the dependence of the rate of CO_2_ methanation on the partial pressures of the reactants and products at different temperatures with 1.5 mg of Ni/Al_2_O_3_ catalyst diluted in SiC, and the results show that the CH_4_ formation is highly dependent on the H_2_ concentration at the CO_2_ partial pressure of 22 kPa (Figure 1), while the dependence on the CO_2_ partial pressure is weak.

Based on the thermodynamic analysis, it was found that suitable low temperatures, pressurization, and a high H_2_/CO_2_ molar ratio are conducive to increasing the yield of the target product (CH_4_). The reaction kinetics analysis showed that too low a temperature would reduce the reaction rate, so the catalyst was required to maintain the activity at a low temperature to reduce energy consumption during the reaction and shorten the reaction time. In addition, the current industrial methanation process usually adopts an adiabatic fixed-bed reactor, and every 1% of CO_2_ conversion would bring about an adiabatic temperature rise of about 60 °C to the system, and even with a larger product gas circulation, it was still difficult to avoid the existence of local hot spots in the catalyst. Even with a large product gas recycling volume, localized hot spots in the bed are still unavoidable. Therefore, methanation catalysts with low-temperature activity and high-temperature stability need to be developed to meet the demands of industrial production. The basic steps of CO_2_ methanation are shown in Table 1.

## 3. Optimization of Reaction Conditions

The CO_2_ methanation reaction is highly sensitive to reaction conditions. Optimizing reaction conditions can significantly improve reaction conversion, selectivity, and product distribution. A complete understanding of these crucial factors can further benefit kinetic evaluation, process optimization, reactor design, and scale-up for industrial applications [47]. In this section, the role of operating conditions such as temperature, pressure, gas hourly space velocity (GHSV), and the H_2_/CO_2_ ratio is extensively discussed.

### 3.1. Effect of Reaction Temperature

The standard enthalpy and the standard Gibbs free energy of the Sabatier reaction (Equation (10)) are ${\Delta H}_{298K}^{\theta }=-165\;\mathrm{kJ}\cdot {\mathrm{mol}}^{-1}$ and ${\Delta G}_{298K}^{\theta }=-142\;\mathrm{kJ}\cdot {\mathrm{mol}}^{-1}$, respectively [47]. Thermodynamically, this is favored at low temperatures (25~400 °C) [48], and the reaction is exothermic. However, the reduction of carbon in the fully oxidized state of CO_2_ to CH_4_ is an eight-electron process with obvious kinetic constraints [49], which requires catalysts and appropriate temperatures to improve the rate and selectivity of the reaction.

**Figure 2:**
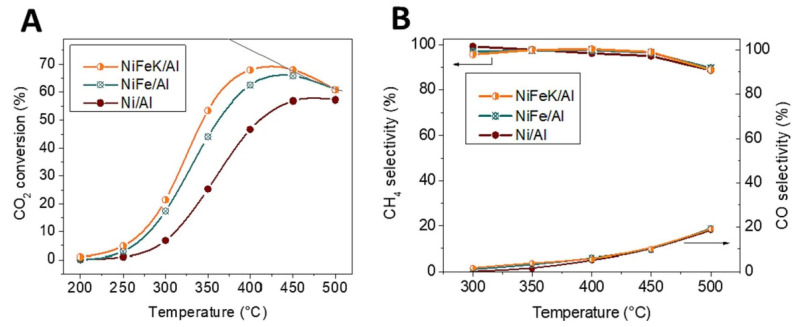
For CO_2_ methanation, catalytic activity at 9 L CO_2_ g^−1^∙h^−1^ and H_2_: CO_2_ = 4: (**A**) CO_2_ conversion; (**B**) %CH_4_ selectivity and %CO selectivity [51]. Copyright (2023) Fuel.

Fan et al. [50] investigated the effect of temperature on the distribution of CO_2_ methanation products by using a micron/nano TiO_2_ catalyst. They found that CH_4_ and H_2_O were the main products at lower temperatures (150~400 °C), while the production of CO gradually increased when the temperature exceeded 450 °C. The reason for this change may be that the RWGS reaction (Equation (1)) favors and dominates at high temperatures. González-Castaño et al. [51] synthesized a series of Ni/γ-Al_2_O_3_, Ni-Fe/γ-Al_2_O_3_, and Ni-Fe-K/γ-Al_2_O_3_ catalysts and evaluated them by methanation experiments. They concluded that the CO_2_ conversion of each catalyst exhibited a volcanic-shaped tendency with an increase in temperature. The reaction rate reached equilibrium conversion at about 450 °C (Figure 2). However, when the temperature was higher than 450 °C, the CO_2_ conversion decreased, obviously due to catalyst deactivation (sinter, agglomeration) and coking [52].

In addition, the low-temperature conversion of CO_2_ methanation on non-noble metal catalysts has been one of the research hotspots in recent years. For example, Zhu et al. [53] prepared a Y_2_O_3_-promoted NiO-CeO_2_ catalyst by designing the oxygen vacancy of the Ni-based catalyst. They introduced the metal oxide Y_2_O_3_, which is incompatible with the support CeO_2_ lattice, to promote the generation of oxygen defects. Under mild conditions (<300 °C), the catalyst was three times more active than the conventional NiO/CeO_2_ catalyst. Ma et al. [54] found that the turnover frequency (TOF) of CO_2_ methanation of Ni/monoclinic-ZrO_2_ was increased by 116% compared with that of Ni/cubic-ZrO_2_ at 240 °C by optimizing the crystal phase of ZrO_2_ in the Ni/ZrO_2_ catalyst, while the activation energy of the reaction was reduced by 24%.

### 3.2. Effect of Reaction Pressure

**Figure 3:**
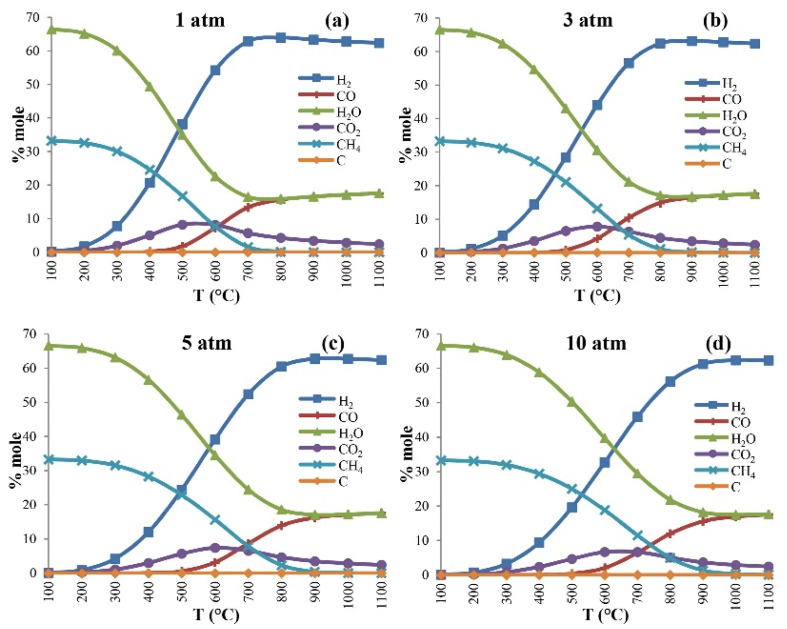
The equilibrium compositions of different cases; (**a**) P = 1, (**b**) P = 3, (**c**) P = 5, and (**d**) P = 10 atm [56]. Copyright (2023) International Journal of Hydrogen Energy.

The CO_2_ methanation reaction is a gaseous reaction in which the number of reactants is reduced. According to Le Chatelier’s law, pressurization is an effective measure to improve the conversion rate. Thermodynamic studies conducted by Ahmad et al. [55] on the Cu-K/Al_2_O_3_ catalyst showed that the equilibrium conversion and CH_4_ yield increased with the increase in pressure and were more sensitive under high pressure. Yarbaş et al. [56] conducted modeling and studied the equilibrium composition of the reaction pressure at 1, 3, 5, and 10 atm, respectively (Figure 3). It was found that the proportion of CH_4_ in the products at all pressures reached its maximum at the lowest temperature (100 °C), and the trend of species changes with temperature was consistent at all pressures. With the increase in pressure, the molar concentration of the reactants (CO_2_ and H_2_) decreased, while the molar concentration of the products (CH_4_ and H_2_O) increased at the same temperature.

Theoretically, higher CO_2_ conversion and CH_4_ selectivity can be achieved under conditions of low temperature and high pressure. However, from an industrial perspective, high-pressure equipment is more dangerous to use, and the low-temperature reaction rate is lower. Therefore, the optimal conditions for CO_2_ methanation are a temperature of 30~500 °C and a pressure of 0.1~0.3 MPa [57].

### 3.3. Effect of Gas Hourly Space Velocity

GHSV is also an important factor affecting the performance of catalysts. For instance, Mihet’s team reported that lower GHSV could improve the catalytic performance of CO_2_ methanation. This is because the lower GHSV directly translates into a longer residence time [58], which increases the contact and reaction time of the catalyst with the reactant feedstock gas. In addition, appropriate GHSV can take away the heat generated in the methanation reaction, and it is not easy to cause excessive temperature and deactivate the catalyst [59].

**Figure 4:**
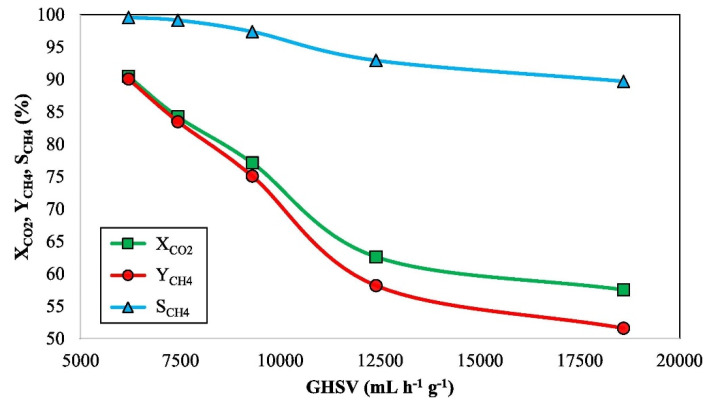
Catalytic performance of 15% Ni/TiO_2_ at 350 °C and atmospheric pressure under different GHSV [50]. Copyright (2022) Energy Conversion and Management.

However, too high GHSV will shorten the residence time of CO_2_ on the catalyst surface, which will reduce the conversion rate. For example, Fan et al. [50] discussed the catalytic performance of methanation on a 15% Ni/TiO_2_ catalyst when the GHSV increased from 6200 to 18,600 mL∙g^−1^∙h^−1^ (Figure 4). It was found that the catalytic performance decreased significantly when the GHSV was 18600 mL∙g^−1^∙h^−1^, which is because not all CO_2_ can be successfully adsorbed and converted into CH_4_ at the active sites of the catalyst at high reaction flow rates. Due to the limited active sites available for the hydrogenation reaction, limiting the conversion of CO_2_ and production of CH_4_.

### 3.4. Effect of H_2_/CO_2_ Ratio

**Figure 5:**
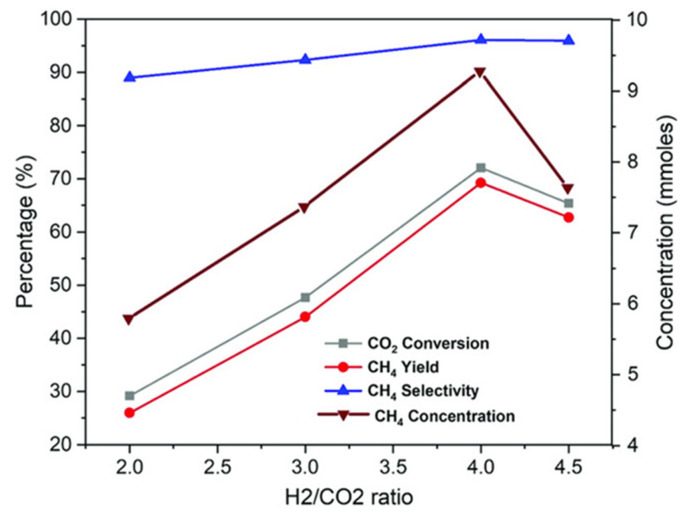
Effect of H_2_: CO_2_ ratio on CH_4_ concentration, CO_2_ conversion, CH_4_ selectivity, and CH_4_ yield [60]. Copyright (2019) Energy Technology.

According to the study [55], the H_2_/CO_2_ ratio has an important influence on the CO_2_ methanation reaction in terms of CO_2_ conversion, CH_4_ yield, and CO and CH_4_ selectivity. For example, Jaffar et al. [60] prepared a 10% Ni/Al_2_O_3_ catalyst and explored the effect of the H_2_/CO_2_ ratio on the CO_2_ methanation reaction in detail by changing the molar ratio of H_2_/CO_2_. The results showed that when the H_2_/CO_2_ ratio increased from 2 to 4, the CO_2_ conversion and CH_4_ selectivity increased from 29.1% and 88.9% to 71.7% and 96.1%, respectively, and the methane concentration increased from 5.8 mmol to 9.3 mmol (Figure 5). However, when the H_2_/CO_2_ ratio reached 4.5, the CO_2_ conversion and CH_4_ yield dropped. This is consistent with the conclusions reached by Aziz et al. [61] and Moghaddam et al. [62]. The possible reason is that when the H_2_/CO_2_ ratio is greater than 4, the excess H_2_ molecules on the catalyst surface compete for CO_2_ adsorption, reducing the active sites available for CO_2_ adsorption.

In addition, the H_2_/CO_2_ ratio also affects the carbon deposition of the catalyst. According to the thermodynamic experiments conducted by Gao et al. [44] on the Ni-based catalyst, when the H_2_/CO_2_ ratio is as low as 2, up to 50% carbon deposition can be observed at 500 °C, while when the H_2_/CO_2_ ratio is equal to or greater than 4, the carbon deposition decreases to 0. Hussain et al. [57] conducted evaluation experiments and thermodynamic studies on the CO_2_ methanation reaction of a metal-free fibrous silica-β zeolite (FS@SiO_2_-BEA) catalyst. It was found that when the H_2_/CO_2_ ratio increased from 1 to 2, the proportion of CH_4_ in the product increased and the proportion of CO decreased. On further increasing the ratio from 2 to 3, the molar fraction of coke formation decreased remarkably, and when the ratio increased to 4, the molar fraction of coke became 0. Based on this observation, it can be concluded that a high H_2_/CO_2_ ratio is conducive to high CH_4_ and no carbon generation.

## 4. Non-Noble Metal Catalysts for CO_2_ Methanation Reaction

### 4.1. Ni-Based Catalysts

The catalysts used for CO_2_ methanation are mainly supported by Group VIII metals, with active metals, including Ru, Rh, Pd, Pt, Fe, Co, Ni, etc. [63]. Among them, Ni-based catalysts have been widely studied in the process of CO_2_ methanation due to their high catalytic activity, high CH_4_ selectivity, abundant reserves, and low price [64].

Ni-based single-atom catalysts have extremely high activity in the activation of C–H bonds, but the carbon deposition generated by C–H bond activation will cover the Ni active sites, resulting in catalyst deactivation [65]. Therefore, it is necessary to further enhance the catalytic activity and CH_4_ selectivity of Ni-based catalysts, such as through the application of supports and promoters, the use of appropriate preparation methods, etc. [66].

#### 4.1.1. Support

The support material affects the activity of the catalyst by improving the dispersion of the active component, adjusting the surface structure of the catalyst, and affecting the strong interaction between the support and the active component [67]. For Ni-based catalysts, conventional oxide supports include Al_2_O_3_, SiO_2_, TiO_2_, CeO_2_, and ZrO_2_, and other supports include zeolites, MOFs, etc. [68].

Al_2_O_3_ has been extensively used for CO_2_ methanation due to its high specific surface area and relatively low cost [69]. Chen et al. [70] prepared the NiMn/Al_2_O_3_ catalyst with various Mn loadings and found that the addition of Mn could improve the dispersion of Ni and facilitate the formation of CH_4_. Moreover, 20Ni2Mn/Al_2_O_3_ has more basic sites, which was conducive to the adsorption and activation of CO_2_, and the CO_2_ conversion (90.5%) was the highest at 250 °C. In this study, Al_2_O_3_ was detected in the calcined catalyst, and Ni species were all present as NiO compounds. The Mn phase is highly dispersed, and no corresponding diffraction peaks were observed using XRD. For the reduced catalysts, Mn species were mainly present in the form of MnO_2_, and Ni became difficult to detect, a phenomenon that suggests that the Mn additives led to a better dispersion of Ni particles in the samples.

**Figure 6:**
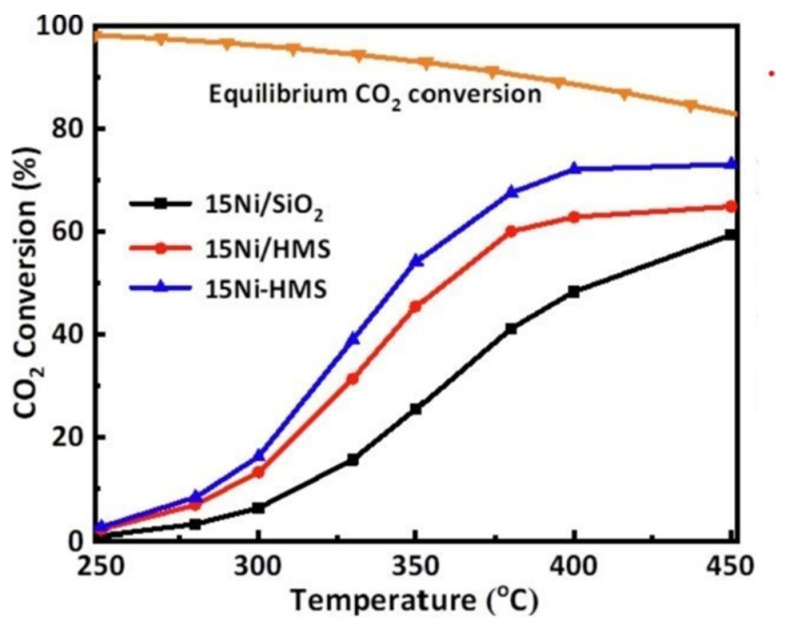
The curves of the CO_2_ conversion versus the reaction temperature over the 15Ni-HMS, 15Ni/HMS, and 15Ni/SiO_2_ catalysts [72]. Copyright (2023) Fuel.

**Figure 7:**
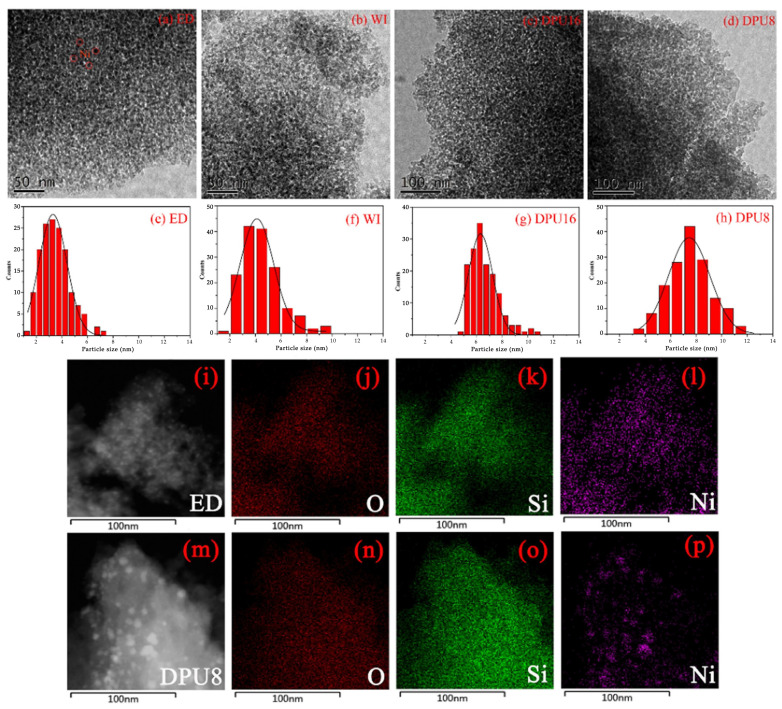
TEM images (**a**–**d**,**i**,**m**) of reduced Ni/SiO_2_ catalysts and the statistics particle size distribution (**e**–**h**). EDS mapping of ED (**j**–**l**) and DPU8 (**n**–**p**) [38]. Copyright (2021) Fuel.

**Figure 8:**
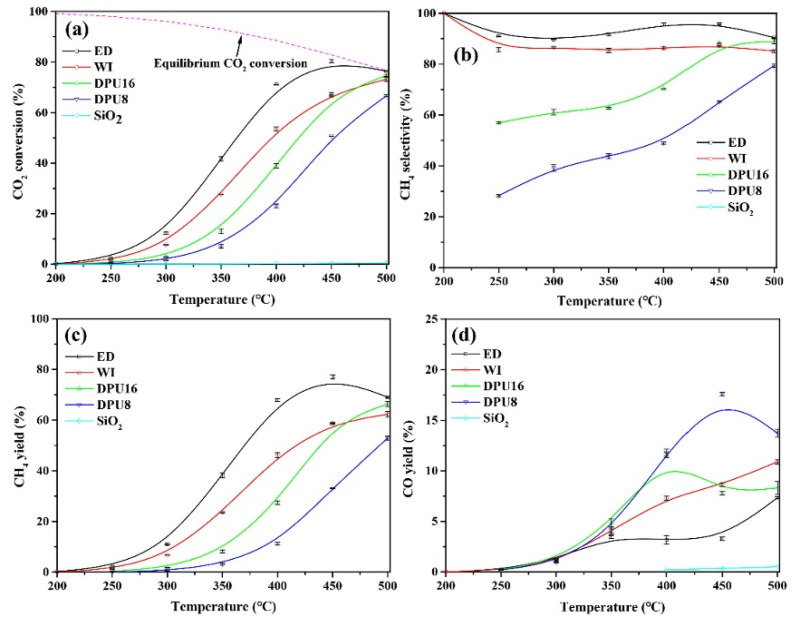
Activity data expressed as CO_2_ conversion and equilibrium CO_2_ conversion (**a**), CH_4_ selectivity (**b**), CH_4_ yield (**c**), CO yield (**d**). Experimental conditions: catalyst mass = 0.1 g, T = 200–500 °C, feed flow = 20 mL/min (2 mL/min CO_2_, 8 mL/min H_2_, 10 mL/min N_2_), GHSV = 12,000 h^−1^ [38]. Copyright (2021) Fuel.

SiO_2_ generally exists in an amorphous state and has easily regulated average pore diameter, specific surface area, and pore volume [71]. Xu et al. [72] prepared the Ni-HMS catalysts using hexagonal mesoporous silica (HMS) as the support by the one-pot method. Compared with Ni/HMS and Ni/SiO_2_ catalysts prepared by traditional methods, the 15Ni-HMS catalyst has the highest CO_2_ conversion and CH_4_ selectivity (99.9%) (see Figure 6). The Ni-HMS mesoporous framework can anchor Ni active sites to inhibit sintering, and Ni particles are highly dispersed, which effectively improves the performance of the catalyst. Wang et al. [38] prepared the catalytic activity of Ni/SiO_2_ catalysts with different Ni particle sizes by different preparation methods and investigated the effect of Ni particle sizes on CO_2_ methanation in the range of 3.5~7.5 nm. As shown in Figure 7, the Ni particles of all samples were spherically supported on SiO_2_ supports, but the catalysts prepared by different methods showed different particle sizes. The catalytic performance of Ni/SiO_2_ catalysts with different Ni particle sizes is shown in Figure 8. It can be seen that the ED catalyst with the smallest nickel particle (3.5 nm) has the highest CO_2_ conversion rate and the best CH_4_ yield in the temperature range of 200~500 °C. The characterization results showed that reducing the size of Ni particles can increase the adsorption of CO_2_ and the number of active sites, thus improving the catalytic activity.

TiO_2_ can promote the dissociation of the C–O bond and CO_2_ hydrogenation activity by electronic interaction with metals [73]. Unwiset et al. [74] prepared Ni/TiO_2_ catalysts with different loads of Ni by the sol–gel method and found that the 20 wt% Ni/TiO_2_ catalyst had the best activity and stability, in which CH_4_ selectivity was almost 100% and CO_2_ conversion was reduced by less than 10% at 420 °C for 72 h. This can be attributed to the fact that increasing Ni helped Ni^2+^ replace Ti^4+^ in the lattice. Moreover, TiO_2_ deformation generates more oxygen vacancies, inhibits the growth of TiO_2_ crystals, reduces the crystal size, and thus increases the specific surface area of the catalyst. Li et al. [75] found that due to the strong metal–support interaction (SMSI) that induced a TiO_2_ coating around Ni nanoparticles, the catalytic activity of CO_2_ methanation of the Ni-based catalyst on the traditional anatase support was inhibited. However, a large amount of Ti^3+^ will be produced after pretreatment of a-TiO_2_ with NH_3_ and H_2_, which can hinder the formation of titanium coating and change SMSI, so that the reactants can be exposed to more Ni species and enhance the catalytic activity of Ni/a-TiO_2_.

**Figure 9:**
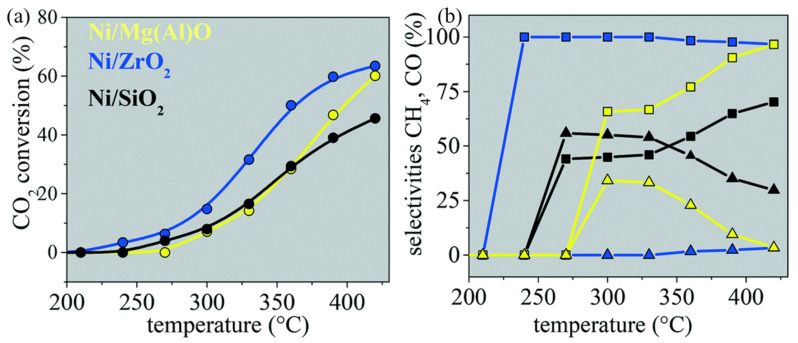
(**a**) CO_2_ conversion versus temperature. The blue, yellow, and black symbols represent Ni/ZrO_2_, Ni/Mg(Al)O, and Ni/SiO_2_, respectively. The experimental conditions are as follows: catalyst mass 200 mg, H_2_/CO_2_ ratio 4:1, flow rate 80 mL∙min^−1^, and pressure 1 atm. (**b**) Selectivity to CH_4_ (squares) and CO (triangles) versus temperature. The catalyst colors and the experimental conditions are the same as in (**a**) [76]. Copyright (2022) Catalysis Science & Technology.

ZrO_2_ has abundant active and basic sites on its surface, good thermal stability, and high porosity [64]. Espino et al. [76] studied the performance of Ni/ZrO_2_, Ni/Mg(Al)O, and Ni/SiO_2_ catalysts in the CO_2_ methanation reaction and found that the Ni/ZrO_2_ catalyst has the highest CO_2_ conversion rate and CH_4_ selectivity (98%), as shown in Figure 9. Due to the insertion of Ni into the ZrO_2_ lattice and its own defects, oxygen vacancy was generated, which improved the adsorption and dissociation of CO on the Ni/ZrO_2_ catalyst.

**Figure 10:**
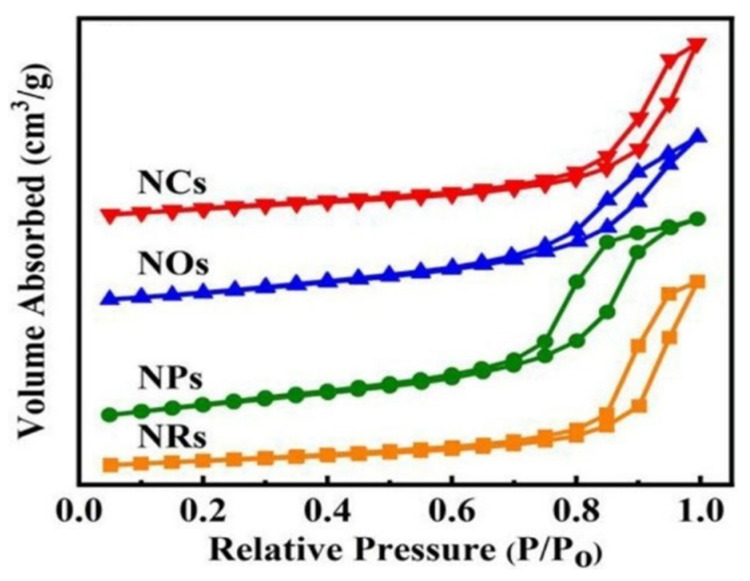
N_2_ adsorption–desorption isotherms of the as-prepared NRs, NCs, NPs, NPs supports [78]. Copyright (2023) Fuel.

CeO_2_ has a high specific surface area and abundant oxygen vacancy [77]. Bian et al. [78] prepared Ni-based catalysts with different CeO_2_ morphologies by the hydrothermal method, namely 5Ni/NPs, 5Ni/NOs, 5Ni/NCs, and 5Ni/NRs. They found that these supports have type IV isotherms, as shown in Figure 10, indicating a rich mesoporous structure from nanocrystal aggregation. They also found that the 5Ni/NPs catalyst had abundant oxygen vacancy, appropriate metal–support interaction, and the best catalytic activity and stability. Moreover, the CO_2_ conversion rate order below 300 °C was 5Ni/NPs > 5Ni/NOs > 5Ni/NCs > 5Ni/NRs, indicating that the morphology of the CeO_2_ support affected the activity of the catalyst. Li et al. [79] compared the catalytic activity of Fe/CeO_2_, Co/CeO_2_, and Ni/CeO_2_ for CO_2_ hydrogenation and found that Ni/CeO_2_ had the highest CO_2_ conversion rate, and the selectivity of Ni/CeO_2_ and Co/CeO_2_ for CH_4_ was almost 100%. In contrast, Fe/CeO_2_ tended to produce CO. The strong interaction between Fe and CeO_2_ can promote the activation of CO_2_ but weaken the activation of H_2_, resulting in poor CO_2_ hydrogenation activity, while the moderate interaction between Ni, Co, and CeO_2_ is conducive to H_2_ activation, thus enhancing the catalytic activity.

Metal–organic frameworks (MOFs) are a kind of crystalline porous material with a periodic network structure composed of metal ions or metal clusters and organic ligands. It has the advantages of porous structure, large specific surface area, regular pore morphology, flexible composition and structure, etc., so it has been widely used in CO_2_ catalytic reduction [80]. Compared with traditional catalysts, MOFs can give catalysts unique morphology, flexible and predictable structure, and uniform element distribution [81]. Lin et al. used a high-surface-area Al-containing metal–organic framework, MIL-53 (Al), as a sacrificial support to obtain Ni-Al_2_O_3_ catalysts with 5 to 20 wt% Ni content. It was found that Ni nanoparticles were highly dispersed in MOF-derived catalysts, and the formation after MOF calcination of nickel aluminate nanodomains was beneficial to improving the activity and stability of the catalyst [82]. Feng et al. pointed out that the smaller the size of Ni particles, the more active sites can be exposed, which is more favorable for improving catalytic activity. However, small particles are prone to aggregation during impregnation, and particle agglomeration can be inhibited by inhibiting the growth of metal nanoparticles through the carrier pores, while MOFs can be used to limit the supported nanoparticles to maintain small sizes in the pores. They synthesized a MOF-derived Ni/CeO_2_ catalyst and obtained highly dispersed ultra-fine Ni nanoparticles by using the constraint effect of the porous structure of MOFs. Calcination at 600 °C increased the number of oxygen vacancies in the Ni/CeO_2_ catalyst, thereby improving the adsorption capacity of CO_2_ and the catalytic performance [83].

#### 4.1.2. Preparation Method

Different preparation methods may affect the grain size of the active component and the metal–support interaction, leading to different catalytic performances. The common catalyst preparation methods include impregnation, co-precipitation, sol–gel, hydrothermal, and deposition-precipitation.

**Figure 11:**
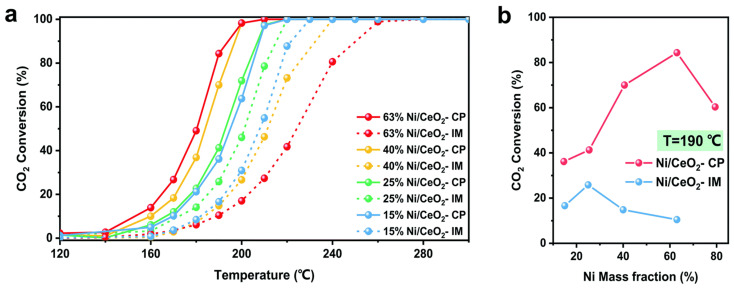
(**a**) CO_2_ methanation activities of the catalysts prepared by the CP/IM method with different Ni loadings at various temperatures (reaction conditions: P = 1.0 atm, SV = 24,300 mL∙g^−1^∙h^−1^, (H_2_/CO_2_) molar ratio = 80), (**b**) comparison of CO_2_ conversion for catalysts with different Ni loadings at 190 °C [84]. Copyright (2022) Catalysis Science & Technology.

The co-precipitation method can be used to prepare non-noble metal oxide catalysts with high metal content, but the performance of the catalyst will be affected by solution concentration, pH, feeding sequence, and stirring intensity. Zhang et al. [84] prepared Ni/CeO_2_ catalysts by a continuous co-precipitation method and found that catalysts prepared by the improved co-precipitation method have better low-temperature CO_2_ methanation activity than catalysts prepared by the impregnation method, as shown in Figure 11.

The active metal of catalysts prepared by the sol–gel method is relatively well-dispersed. Moghaddam et al. [85] used a new sol–gel method without surfactants to synthesize an Ni/Al_2_O_3_ catalyst with 15~30 wt% Ni content. It was found that the specific surface area of the catalyst was large, up to 269.2 to 297.3 m^2^∙g^−1^, and the Ni is easier to reduce due to the weak interaction between NiO and Al_2_O_3_. Moreover, 30Ni/Al_2_O_3_ had the best catalytic performance at 350 °C, the CO_2_ conversion was 73.98%, and the CH_4_ selectivity was 99%. The catalyst samples after 700 °C calcination were mainly cubic nickel aluminate spinel, and no NiO peaks were detected even at higher Ni content, suggesting that Ni is highly dispersed.

The catalyst prepared by the hydrothermal method has good dispersion, uniform distribution, and light particle agglomeration and is commonly used to synthesize catalysts with controllable morphology. Liu et al. [86] compared the performance of Ni/SiO_2_ catalysts prepared by impregnation, the sol–gel method, and hydrothermal methods. It was found that the Ni particle size on the catalyst prepared by the hydrothermal method was the smallest and the dispersion was the best, and there were more active sites on the surface of the catalyst, resulting in better catalytic performance.

In addition to those mentioned above, there are several preparation methods. For example, the active components of the catalyst obtained by impregnation can be evenly distributed in the pores of the support. However, it may cause the migration of active components during the drying process [87]. The deposition–precipitation method can improve the utilization rate of active components but requires a high specific surface area of the support, and the metal particles generated are larger and the uniformity is low [88].

#### 4.1.3. Promoter

Adding various kinds of metal promoters to Ni-based catalysts can improve the dispersion of active components on the surface of the catalyst to regulate the relationship between the active components and support, thereby improving the activity and stability of catalysts. The promoters of Ni-based catalysts mainly include alkaline earth metals, transition metals, and rare earth metals [89].

Liu et al. [90] modified Ni/CeO_2_ by doping alkaline earth metals (Mg, Ba, Sr, and Ca) and found that the activity of Ni/CeO_2_ was improved. The activity order of catalysts was Ni/Ca_0.1_Ce_0.9_O_x_ > Ni/Sr_0.1_Ce_0.9_O_x_ > Ni/Mg_0.1_Ce_0.9_O_x_ > Ni/Ba_0.1_Ce_0.9_O_x_ > Ni/CeO_2_, and the CO_2_ conversion of Ni/Ca_0.1_Ce_0.9_O_x_ was 75% at 290 °C, CH_4_ selectivity was 99%. The chemical characterization of catalysts showed that adding alkaline earth metal oxides was beneficial to improve the dispersion of Ni, and there were more oxygen vacancies on the surface of Ni/M_0.1_Ce_0.9_O_x_, which was related to medium basic sites on the surface of catalysts.

**Figure 12:**
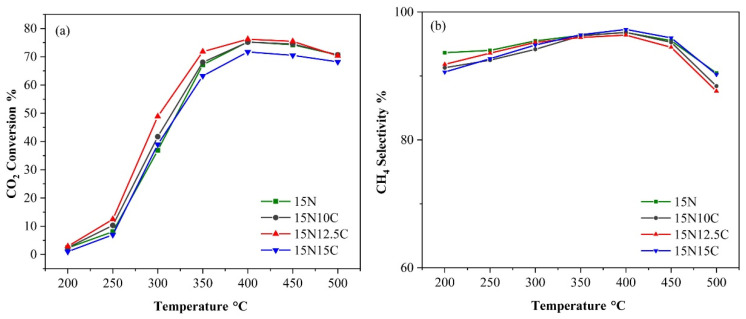
Effect of cobalt loadings on (**a**) CO_2_ conversion and (**b**) CH_4_ selectivity of the 15Ni-Co-Al_2_O_3_ catalysts calcined at 500° with (GHSV = 9000 mL∙g_cat_^−1^∙h^−1^, (H_2_/CO_2_) molar ratio = 4) [92]. Copyright (2021) International Journal of Hydrogen Energy.

The transition metals Ti, Mn, Fe, Co, and Cu have unique acid–base and reduction properties. Akash et al. [91] compared the CO_2_ methanation reaction on 17 wt% Ni/γ-Al_2_O_3_ and 17 wt% Ni_3.2_Fe/γ-Al_2_O_3_ catalysts and found that adding Fe could improve the stability of Ni^0^, and Fe inhibited the formation of CO^*^ or the adsorption of CO. The 17 wt% Ni_3.2_Fe/γ-Al_2_O_3_ catalyst had a higher CO_2_ conversion of 42%. Shafiee et al. [92] modified Ni-Al_2_O_3_ by adding different contents of Co and found that the activity of catalysts was improved. As shown in Figure 12, increasing the content of Co could increase the CO_2_ conversion. The 15Ni-12.5Co-Al_2_O_3_ has the best catalytic performance, with CO_2_ conversion at 76.2% at 400 °C and CH_4_ selectivity at 96.39%.

Reihaneh Daroughegi et al. [93] studied the effect of adding Zr, Ce, La, and Mo on the performance of Ni-Al_2_O_3_ catalysts and found that the catalysts with added promoters had higher Ni content and better reducibility at high temperatures and could also improve the interaction between Ni and Al_2_O_3_. Among them, the 25Ni-5Ce-Al_2_O_3_ catalyst had the smallest Ni crystal size, and Ni could be highly dispersed. The addition of Zr and Ce could promote the formation of oxygen vacancies on the catalyst, thereby enhancing the adsorption capacity of CO_2_.

### 4.2. Co-Based Catalysts

Co and Ni belong to the group VIII elements; the activity of hydrogenation reactions is strong for both of them. However, Co catalysts have a poor ability to catalyze the water–gas shift (WGS) reaction. On the other hand, in the CO_2_ hydrogenation reaction, Co can not only be used as a promoter for Ni-based catalysts but also as an active component itself [94,95]. Because of the weak activity of Co in the WGS reaction, the amount of CO_2_ adsorbed on the surface of Co is very low, resulting in a small C/H ratio on the surface and the easy formation of CH_4_, which has the characteristics of less carbon deposition and high selectivity. Considering the characteristics of cost, applicability, stability, and activity, the Co catalyst has certain research significance.

#### 4.2.1. Supports

The support has a crucial influence on the activity of Co-based catalysts. Through the strong interaction between the carrier and Co particles, the redox properties and adsorption properties of the catalyst are affected, and the performance of the catalyst is changed [49]. At present, Co-based catalysts mostly use metal oxides such as ZrO_2_, Al_2_O_3_, SiO_2_, TiO_2_, zeolite molecular sieves, and other polymer porous materials.

Among them, the most studied Co/Al_2_O_3_ and Co/ZrO_2_ catalysts have the closest and highest CH_4_ yields. The Co/ZrO_2_ catalyst has higher activity, CO_2_ conversion, and stability when it is close to equilibrium. Li et al. loaded 10 wt% Co on ZrO_2_ by the impregnation method, and the activity remained after 300 h and the selectivity of CH_4_ reached 99.9% [96]. Al_2_O_3_ has a wide range of sources and is easy to prepare. The FeCo/Al_2_O_3_ catalyst exhibits excellent catalytic performance under mild reaction conditions (280 °C, 0.2 MPa), whose reaction rate was 8.7 μmol g_cat_^−1^∙s^−1^, under the synergistic effect of Al_2_O_3_ support and Fe, the yield is significantly higher than that of Co/Al_2_O_3_ catalyst (1.68·10^−3^ mol∙g_cat_^−1^∙min^−1^) [97]. In this study, researchers used in situ XRD to follow the intermediate-state products of Co and Fe. As for samples with equal amounts of FeCo, there was an apparent metallic Fe^0^ phase at 600 °C, and Fe_3_O_4_ and CoFe_3_O_4_ were detected. Although there was no CoO/Co phase detected, the presence of metallic Fe^0^ definitely indicated the existence of interaction and the improvement of reducibility.

Lauterbach et al. [98,99] compared the interaction of SiO_2_, Al_2_O_3_, and TiO_2_ supports with cobalt nanorods (CoNR) materials. In the kinetically controlled temperature range (150~250 °C), the CoNR/TiO_2_ catalyst exhibited higher catalytic activity than the other two catalysts. Whereas, in the temperature range of mass transfer control (>300 °C), CoNR/Al_2_O_3_ exhibits significantly enhanced activity due to the good thermal conductivity of Al_2_O_3_. Compared with CoNR/TiO_2_ and CoNR/Al_2_O_3_, CoNR/SiO_2_ shows no change in catalytic activity compared with pure CoNR, indicating that the promotion effect of SiO_2_ support was not obvious.

In the direction of zeolite material support, Nadia et al. [100] prepared a special methanation catalyst by using Co-based layered zeolitic imidazolate framework (ZIF-L) material under an argon atmosphere, that is, fixed on highly porous nitrogen Co nanoparticles in a doped carbon matrix. The specific activity of the catalyst at 350 °C is 22.3 mol_CH4_∙g_cat_^−1^∙min^−1^, which is significantly better than more conventional ZIF-67-like catalysts (11.7 mol_CH4_∙g_cat_^−1^∙min^−1^). This is due to the stability of Co nanoparticles (~20 nm) and the abundant basic active sites associated with nitrogen doping in ZIF-L-prepared catalysts. What is more, this new catalyst has not been observed to be deactivated within 60 h, which shows the high stability of the catalyst.

#### 4.2.2. Preparation Method

**Figure 13:**
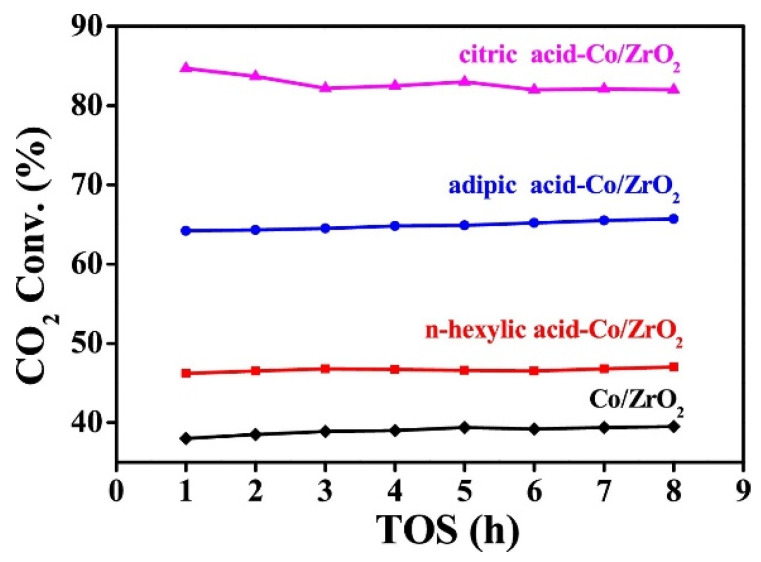
The catalytic performance of n-hexylic acid, adipic acid, and citric acid-assisted Co/ZrO_2_ catalysts and the benchmark Co/ZrO_2_ catalyst. Conditions: molar ratio of H_2_/CO_2_ = 4/1, GHSV = 7200 mL∙g^−1^∙h^−1^, P = 3 MPa, T = 400 °C [95]. Copyright (2019) Applied Catalysis B-Environmental.

In order to obtain a highly dispersed catalyst, the preparation method is also an important part of the activity of the catalyst. On the idea of preparing Co/ZrO_2_ catalyst by the impregnation method, Li et al. [95] adopted the method of organic acid-assisted preparation to obtain a highly dispersed Co/ZrO_2_ catalyst. The loading of Co in the carrier was only 2 wt%, but the CO_2_ methanation reaction exhibits excellent catalytic performance. Among many organic acids, the Co/ZrO_2_ catalyst prepared by citric acid has the best catalytic activity, and the conversion of CO_2_ is as high as 85%, see in Figure 13. At the same time, citric acid-assisted preparation of Co/diatomite catalysts is also an effective method to improve the performance of the catalyst. The CO_2_ conversion is 73%, the CH_4_ selectivity is 96%, and it shows good stability in the long-term reaction [101]. The assistance of organic acids can effectively improve the metal dispersion of Co catalysts and thus obtain more active sites for CO_2_ adsorption and catalytic hydrogenation.

**Figure 14:**
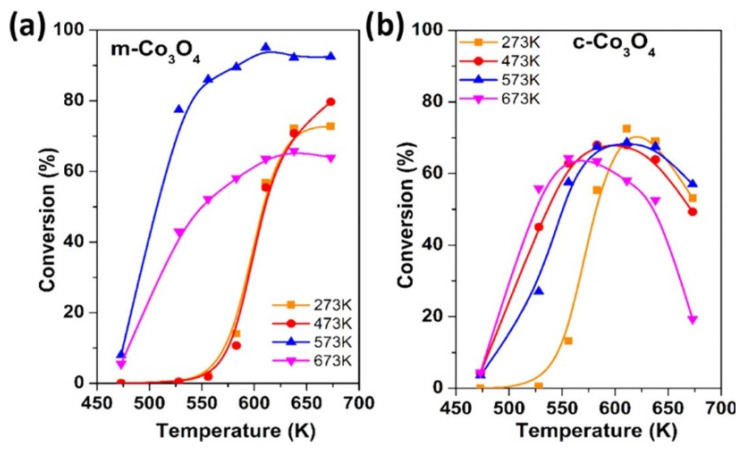
CO_2_ conversion over (**a**) m-Co_3_O_4_ and (**b**) c-Co_3_O_4_ reduced at 273, 473, 573, and 673 K [102]. Copyright (2021) Journal of Physical Chemistry C.

Anastasiia et al. [102] synthesized a mesoporous m-Co_3_O_4_ catalyst with a BET surface area of 95 m^2^∙g^−1^ using the co-precipitation method. And found that the m-Co_3_O_4_ catalyst was more active than the commercial c-Co_3_O_4_ (BET surface area of 15 m^2^∙g^−1^) catalyst and had better thermal stability at high temperatures, see in Figure 14. This study shows that in the CO_2_ methanation reaction, factors such as the morphology of the catalyst body and surface and the existence of defect sites directly affect the catalytic performance and reaction mechanism of the catalyst [48].

Compared with the impregnation method, the catalysts prepared by the co-precipitation method have a stronger interaction between Co oxide and Ce. For the preparation of Co/CeO_2_ catalysts, a moderate calcination temperature is a reasonable condition to achieve high CASA (catalytically active surface area), but when the calcination temperature of co-precipitated and impregnated catalysts is 500~700 °C, the total number of basic sites will significantly decrease [103]. DFSP (double flame spray pyrolysis) enables the control and separation of the particle formation process of different catalyst components and can be used to evaluate the effects of active metals, support materials, dopants, and promoters, as well as particle size distribution and porosity. Max Gäßler et al. [104] used DFSP technology to support Co nanoparticles with the same particle size distribution on SiO_2_, TiO_2_, and SiO_2_-TiO_2_ mixtures, and obtained the activity, selectivity, and deactivation behavior of the supported Co catalysts from the support materials main conclusions.

#### 4.2.3. Promoter

**Figure 15:**
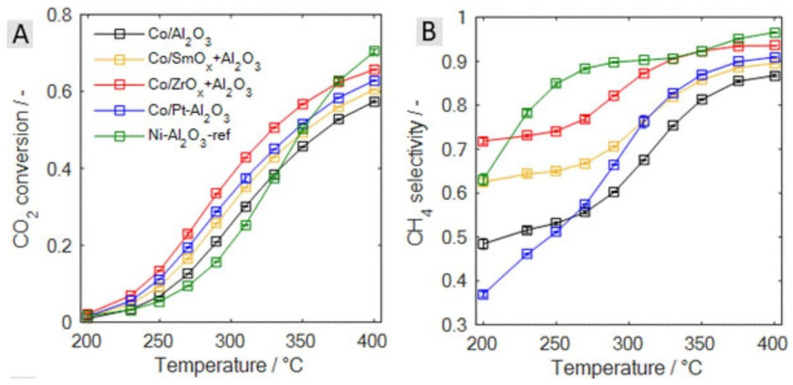
Results of the CO_2_ methanation experiments for all studied catalysts. (**A**) CO_2_ conversion vs. temperature; (**B**) CH_4_ selectivity vs. temperature. Conditions: total pressure 1 bar, total flow rate 50 mLS∙min^−1^ composed of 4/1/5 H_2_/CO_2_/Ar, 25 mg of catalyst [105]. Copyright (2021) ChemCatChem.

Jakob Stahl et al. [105] used the DFSP method to synthesize catalysts with different promoters with the same size distribution and dispersion to study the co-catalytic effect of Pt, ZrO_x,_ SmO_x_, and other promoters on Co-based catalysts. Among them, ZrO_2_ and Pt performed significantly better than SmO_x_, as shown in Figure 15. With the introduction of the Pt promoter, the H_2_ absorption rate and the corresponding metal surface area are significantly increased, while the CO_2_ adsorption capacity is similar to that of the Co catalyst without the promoter.

In order to achieve high-efficiency catalysis of CO_2_ methanation by non-noble metal catalysts at low temperatures (<200 °C), Tu et al. [106] found that by adding Zr to amorphous Co catalysts, the low-temperature catalytic effect was similar to that of noble metals. Since the Zr promoter can expand the surface area of the catalyst, adjust the valence state of the surface atoms of the catalyst, and combine with the rich surface defects and inherent active sites brought about by the amorphous nature of the catalyst itself, high-efficiency catalysis at low temperatures is realized.

The addition of an Mn promoter can significantly improve the performance of Co-based catalysts, which not only promotes the overflow of hydrogen from active Co sites to spinel supports but also leads to a significant increase in the catalytic active surface area of Mn-modified catalysts [107].

### 4.3. Other Catalysts

In addition to Ni-based and Co-based catalysts, supported Fe-based catalysts are also commonly used as active catalysts for CO_2_ hydrogenation. Because traditional Ni-based catalysts are inactivated by sintering under high-temperature conditions, Fe-based catalysts are non-toxic, cheaper, and easier to obtain. They have a higher specific surface area and can react at lower pressures [97].

**Figure 16:**
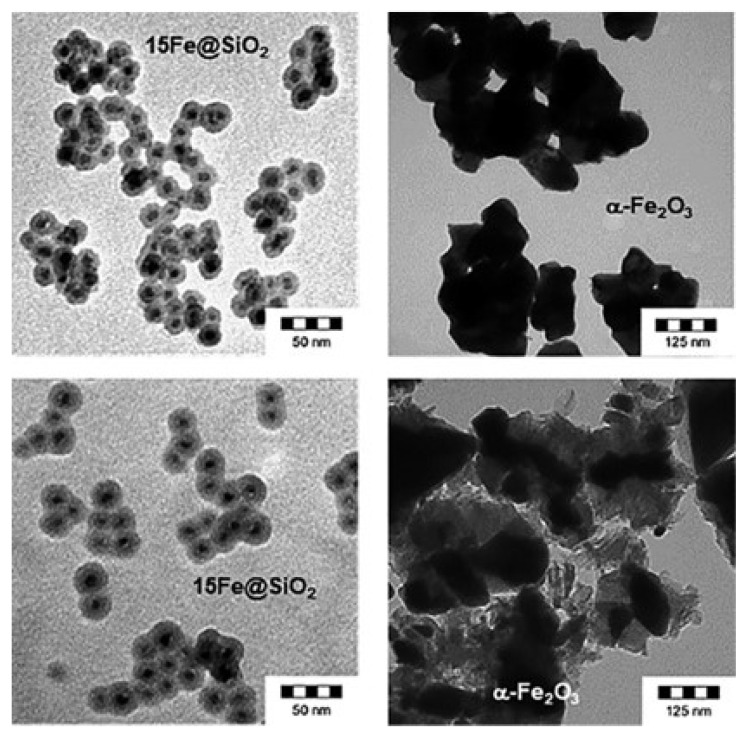
Preparation (**top**) and consumption (**bottom**) of TEM micrographs of α-Fe_2_O_3_ and 15Fe@SiO_2_ sample preparation [109]. Copyright (2020) Chemie Ingenieur Technik.

Similar to the above, the catalytic performance of Fe-based catalysts depends to a large extent on synthesis methods, such as impregnation, coprecipitation, sol–gel, etc. [108]. For instance, Güttel et al. [109] prepared α-Fe_2_O_3_ catalysts, Fe-based catalysts supported by SiO_2_ (15Fe/SiO_2_), and core–shell catalysts (15Fe@SiO_2_), and compared their CO_2_ methanation activity. The α-Fe_2_O_3_ and 15Fe/SiO_2_ catalysts were all prepared by the impregnation method, while the core–shell 15Fe@SiO_2_ catalyst was obtained by the adaptive inverse microemulsion method [110]. The experimental results show that under the reaction condition of 400 °C, 1 bar, with a space velocity of 52,000 h^−1^(69.2 mmol(CO_2_)/mol(Fe)/s) and H_2_/CO_2_ = 4, the CH_4_ generation rate of the α-Fe_2_O_3_ and 15Fe/SiO_2_ catalysts is similar, which is 0.25 mmol_(CO2)_∙(mol_(Fe)_∙s)^−1^ and the 15Fe@SiO_2_ catalyst has better stability. Combined with the characterization technology, it was found that there is no carbon deposition on the 15Fe@SiO_2_ catalyst with a core–shell structure after reaction for 17 h without the reaction process of hot sintering, and the core–shell structure is complete [109]. However, the depletion of α-Fe_2_O_3_ samples is subjected to breakage or wear, and the stability is not high [111]. They pointed out that the methanation activity of the 15Fe@SiO_2_ catalyst needs to be improved, but its carbon deposition is about 300 times less than that of the α-Fe_2_O_3_ catalyst and 32 times lower than that of impregnated Fe-based catalysts, their TEM plots are shown in Figure 16. Moreover, the stability of 15Fe@SiO_2_ catalysts is significantly improved [109].

In addition, TiO_2_, SiO_2_, ZrO_2_, and Al_2_O_3_ are commonly used as metal oxide supports for catalysts in methanation reactions. Toemen et al. [112] synthesized a novel metal oxide (Ru/Fe/Ce) on the γ-Al_2_O_3_ catalyst by the impregnation method and investigated the methanation activity under atmospheric pressure. They compared the various properties of Ru/Fe/Ce γ-Al_2_O_3_ catalysts at different calcination temperatures. It was found that the Ru/Fe/Ce (5:10:85)/γ-Al_2_O_3_ catalysts calcined at 900 °C had a higher specific surface area (73.87 m^2^∙g^−1^) than those calcined at 1000 °C (51.07 m^2^∙g^−1^) and 1100 °C (22.30 m^2^∙g^−1^). The increase in calcination temperature will reduce the specific surface area and pore volume of the catalyst. When the temperature rises to 1100 °C, the larger the pore size and the smaller the pore volume, the lower the catalytic activity. Therefore, the 10 g Ru/Fe/Ce (5:10:85)/γ-Al_2_O_3_ catalyst calcined at 1000 °C has a higher catalytic activity, the CO_2_ conversion rate is increased to 100%, and the CH_4_ yield is 90.5%. In addition to the above oxides, zeolite can also be used as the carrier of Fe-based catalysts, and the interaction between the two can improve the dispersion of Fe species. For instance, using zeolite as the support of Fe-based catalysts, Franken et al. [113] studied the impregnation method to prepare three catalysts with different loading loads (1, 5, and 10 wt%) and analyzed the zeolite structure with relevant characterization. It was found that the impregnation process and calcination temperature had a great impact on the stability of zeolite. The results show that the reaction rate is 42 mmol_(CO2)_∙(mol_(Fe)_∙s)^−1^ and the selectivity of CH_4_ is 76% with a loading of 1 wt% at 300 °C and 10 bar. When the loading of Fe is high, it tends to form more and more high-carbon hydrocarbons in Fischer–Tropsch reaction products under increased pressure. In addition, the catalysts with high Fe loading are more likely to destroy the zeolite structure and form Fe_3_C around the support, which affects the selectivity of CH_4_. Therefore, avoiding the formation of the Fe_3_C phase is crucial for the high selectivity of CH_4_.

Promoters are also an important part of the catalyst and have an important impact on the activity of the catalyst. For example, Kureti et al. [114] prepared a series of Fe-based catalysts with different Mg contents, deeply explored the influence of promoters on the methanation reaction of Fe-based catalysts, and found that Mg as a promoter could further enhance catalyst activity by improving the basicity of the catalyst surface and greatly improving the CO_2_ conversion rate. The results showed that when the load of Mg is 2 wt%, the highest CH_4_ yield is 32% and the selectivity is 65%. In addition, Landau et al. [115] studied the effects of TiO_2_, SiO_2_, ZrO_2_, and MnO on the hydrogenation of CO_2_ in the presence of the surfactant cetyl trimethyl ammonium bromide (CTAB). The results showed that TiO_2_ at 20 wt% increased the rate of RWGS and FTS. The selectivity of CO was decreased and that of CH_4_ was increased, which is conducive to the methanation reaction.

In addition to the Fe-based catalyst mentioned above, the Mo-based catalyst has good sulfide performance and can be used for methanation reactions [116]. Qin et al. [117] found that Mo/Al_2_O_3_ catalysts are widely used in chemical production, but the interaction between Mo and Al_2_O_3_ is not strong enough under high-temperature methanation reactions, and the dispersion of MoS_2_ supported by Al_2_O_3_ is poor, which makes it easy to sinter in the reaction. The interaction between Mo and CeO_2_ is stronger than that between Mo and Al_2_O_3_. The Mo phase supported on CeO_2_ tends to exist in the form of monolayer MoS_2_, but highly dispersed MoS_2_ is unstable. CeO_2_/Al_2_O_3_-supported MoS_2_ catalysts have high dispersion and excellent high-temperature stability. The effects of the roasting method and precipitation method on the preparation of CeO_2_ on the Mo-based catalyst are compared by relevant characterization methods, and the results show that the catalytic activity and stability of the CeO_2_ support prepared by the roasting method are higher [118]. Wang et al. [119] prepared MoO_3_/CeO_2_ catalysts by the impregnation method. They studied the effect of MoO_3_ loading on methanation catalytic performance and found that the CO conversion rate reached its maximumwhen 5 wt% MoO_3_ was added to CeO_2_ support. Wang et al. [120] reported that MoP catalysts were widely used in CO hydrogenation reactions, and a series of MOP-x (x = P/Mo ratio from 1 to 5) catalysts were prepared by pyrolysis of phytic acid (PA)-derived Mo complexes in H_2_ atmosphere. The physicochemical properties and catalytic activity of MoP catalysts were studied, and the characterization results further confirmed that the Mo^δ+^ site of MoP catalysts is the active site of methanation reactions, and its content on the surface of MOP-x catalysts determines the catalytic activity of the catalysts. In addition, Cu-based catalysts are also used as catalysts for CO_2_ hydromethanation. Gabor Varga et al. [121] prepared an efficient Cu–Co bimetallic catalyst for CO_2_ methanation through a spinel oxide precursor system and found that Cu_0.4_Co_2.6_O_4_ showed high activity and high CH_4_ selectivity (65–85%) in the temperature range of 250~425 °C. However, Cu-based catalysts are mostly used for CO_2_ hydrogenation electrocatalytic conversion or CO_2_ hydrogenation to methanol.

## 5. Reaction Mechanisms

**Figure 17:**
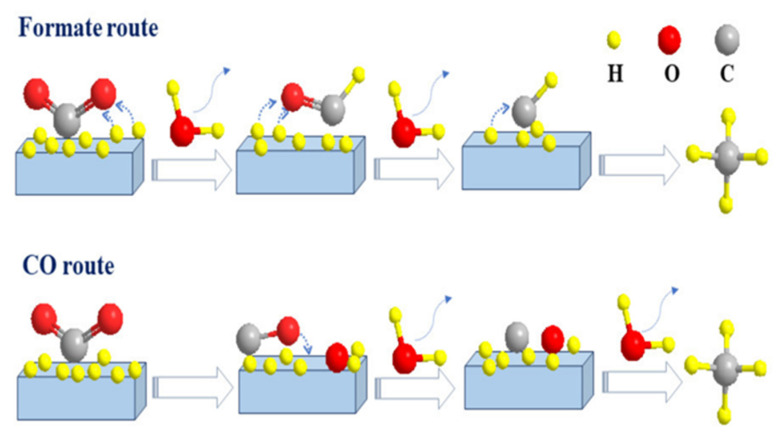
Different CO_2_ methanation routes: formic acid route and CO route [68]. Copyright (2022) Catalysts.

An in-depth understanding of key intermediates and reaction mechanisms is critical for the design of catalysts [122]. Many researchers have elucidated possible CO_2_ methanation mechanisms through in situ FTIR, mass spectrometry (MS) techniques, and density functional theory (DFT) calculations. Although there is much debate about the intermediates and different reaction pathways for the generation of CH_4_, these mainly include the following two pathways: (1) The CO pathway, also known as CO_2_ dissociation methanation: chemisorbed *CO_2_ can dissociate into *CO and *O. The formed *CO can be further dissociated into carbon (*C) and then hydrogenated to CH_4_ by desorption of the dissociated H_2_ on the metal particles from the catalyst surface, while *O can react with hydrogen to form H_2_O. (2) Formic acid pathway: The formic acid substance is the main intermediate product formed in the CO_2_ methanation reaction, also known as CO_2_-associated methanation: Chemisorbed *CO_2_ is first converted to didentate (HCOO*), then to formic acid (HCOOH), and then to CH_4_ [48,123,124,125,126,127,128]. The possible reaction pathways are shown in Figure 17. In addition to the above two pathways, there are other pathways, such as direct methanation of CO_2_ [129], intermediates of both CO and formic acid, etc.

### 5.1. CO Intermediate Mechanism

The CO pathway dissociates CO_2_ from CO prior to methanation, and in the subsequent reaction, CO is converted to CH_4_ by reacting with H_2_ [68]. Although the H-assisted CO dissociation mechanism has been widely elucidated, there are differences in C–O bond breaking. Through DFT calculations and microdynamics studies, Li et al. [130] studied the cleavage of C–O bonds through CHO intermediates at low Co (0001) coverage, which mainly controls the methanation rate of CO. The mechanism is independent of the functional groups considered and the presence of graphitic carbon and may also be applicable to other Co surface structures, which may be used to design improved CO hydrogenation catalysts. Liu et al. [131] used the DFT calculation method to determine the internal reaction mechanism of CO_2_ methanation on Ni/CeO_2_ catalysts. It was found that CO_2_ methanation on Ni/CeO_2_ catalysts may be the conversion of CO_2_ to CO by the RWGS, which then converts it to CH_4_ through the same pathway as CO methanation. Because of the high energy barrier, there is no obvious advantage to forming formates or directly cracking C–O bonds. CO_2_* → HOCO* → CO* → HCO* → H_2_CO* → CH_2_* → CH_3_* → CH_4_* is the optimal reaction pathway. C–O bond breaking of H-assisted H_2_CO* is the rate-limiting step in the RWGS + CO-hydro pathway and plays a crucial role in the CO_2_ methanation process. Bentrup et al. [132] studied the hydrogenation of CO_2_ to CH_4_ using Ni-sepiolite and Ni-marble as catalysts. On the Ni-sepiolite catalyst, the support material cannot adsorb CO_2_, and the dissociation adsorption of CO_2_ in the presence of H_2_ is observed to be activated by the dissociation of H atoms on NiO particles. Therefore, linear and bridged CO on NiO are identified as intermediates, where preferentially linearly bonded CO is hydrogenated to CH_4_. Henriques et al. [133] performed multiple infrared measurements of zeolitride under CO_2_/Ar or CO_2_/H_2_/Ar conditions. In the absence of H_2_, CO_2_ is almost not adsorbed by acidic zeolites, but in the presence of H_2_, formates and carbonyl groups can be detected. The results show that the CO_2_ methanation pathway is not formed by carbonate but by the dissociation of formate on Ni, forming adsorbed CO. Moreover, the results obtained using FTIR show the presence of bidentate carbonates on Ni^2+^ in the remaining NiO, which are highly thermally stable, and these bulk carbonates appear to be “spectator” species that do not participate in the methanation reaction. Therefore, CO is considered to be the main intermediate, and its dissociation is the determining step of CO_2_ methanation. Similarly, Peebles et al. [134] investigated the methanation and dissociation of CO_2_ on Ni (100) surfaces. The formation and activation energies of CH_4_ and CO are 88.7 kJ∙mol^−1^ and 72.8~82.4 kJ∙mol^−1^, respectively. Under the same reaction conditions, the activation energy and reaction rate of CO_2_ to CH_4_ are very close to the activation energy and reaction rate of CO methanation, so CO is an intermediate of CO_2_ methanation [134]. Jacquemin et al. [135] studied the reaction mechanism of CO_2_ methanation on an Rh/γ-Al_2_O_3_ catalyst. CH_4_ is the only hydrocarbon product observed in MS. They think that CO_2_ dissociates the CO, and oxygen adsorbs on the surface of the catalyst. In situ FTIR experiments demonstrated that the formation of CO (ads) is confirmed by linear RH-CO (2048 cm^−1^), Rh^3+^-CO (2123 cm^−1^), and GEM-dicarbonyl Rh-(CO)_2_ (2024 and 2092 cm^−1^), and CO_2_ with dicarbonyl and CO associated with Rh_2_O_3_ react most actively with hydrogen.

### 5.2. Mechanism of Formic Acid Intermediates

Muroyama et al. [136] studied the mechanism of CO_2_ methanation on Ni/Al_2_O_3_ and Ni/Y_2_O_3_ catalysts using in situ FTIR spectroscopy. For Ni/Al_2_O_3_, CO_2_ is dissociated and adsorbed on Ni particles. By introducing hydrogen into the atmosphere, linear and bridged CO adsorbents are converted to nickel carbonyl hydride or formyl substances, which are then hydrogenated into CH_4_. Compared with Ni/Al_2_O_3,_ formic acid and bridging CO are formed on Y_2_O_3_ support and Ni particles, respectively, and the reaction on Ni/Y_2_O_3_ is mainly carried out by forming formic acid adsorption. The CO adsorbates over the Ni particle are hydrogenated to CH_4_ via the formation of nickel carbonyl hydride and/or formyl species. The hydroxyl group of Y_2_O_3_ facilitates the conversion of carbonates and bicarbonates into formates, and formic acid intermediates are located on Y_2_O_3_, so Ni and Y_2_O_3_ can directly participate in the reaction to make the catalyst more active overall [137].

**Figure 18:**
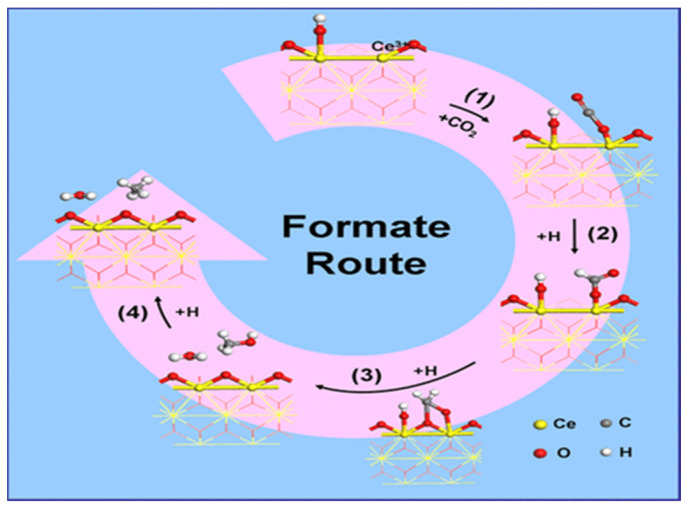
Schematic diagram of CO_2_ methanated formic acid on Ru/CeO_2_ catalyst [138]. Copyright (2016) Journal of the American Chemical Society.

Wang et al. [138] investigated the active site-dependent mechanism of CO_2_ methanation catalyzed by the Ru/CeO_2_ catalyst, using XANES, IR, and Raman to study the formation process of Ce^3+^, surface hydroxyl, and oxygen vacancy in Ru/CeO_2_, and clearly revealed their structural evolution under reaction conditions. Steady-state isotope transient kinetic analysis (SSITKA) type in situ drift infrared spectroscopy confirmed that in the presence of Ru/CeO_2_, these substances are involved in the catalytic process of the formic acid route, and oxygen positions catalyze the dissociation of formic acid to methanol, which is the rate-determining step, and eventually form methane (as shown in Figure 18).

Wang et al. [126] studied the adsorption and methanation of CO_2_ on an Ni/Ce_0.5_Zr_0.5_O_2_ catalyst by in situ FTIR spectroscopy. They found that CO_2_ is more likely to be adsorbed at the surface oxygen site near Ce(III), and the unidentate carbonates formed on Ce(III) are easy to hydrogenate. Formate is the main intermediate product in the reaction, which is formed by the hydrogenation of hydrogen carbonate and unidentate carbonates. Schild et al. [139] studied CO_2_ methanation on an Ni-Zr catalyst by in situ FTIR. It was observed that CH_4_ production increased with the depletion of the formic acid signal, and it was concluded that formate is a necessary intermediate for methane production.

## 6. Conclusions and Outlook

Non-noble metal catalysts have excellent catalytic activity for CO_2_ methanation, are low priced, and are conducive to wide application in industry. Therefore, this paper reviews the latest progress of non-noble metal catalysts in CO_2_ methanation from four aspects, namely CO_2_ methanation thermodynamics and kinetics, non-noble metal catalysts, the effect of reaction conditions, and reaction mechanisms. In this work, the kinetics and thermodynamic characteristics of CO_2_ methanation are explained, and it is clear that the reaction is an exothermic and volumetric shrinkage reaction, so low temperature and high pressure are more conducive to the reaction. Secondly, the influences of supports, promoters, and preparation methods on the catalytic performance of CO_2_ methanation of Ni-based, Co-based, and other catalysts were summarized. It is found that the catalytic performance is affected mainly by reducing the particle size of the active metal, increasing the dispersion of the active metal, improving the strong metal–support interaction, and regulating the number of oxygen vacancies and the number of basic sites. In addition, the reaction conditions also affect the catalytic activity; suitable reaction conditions are beneficial to improving catalytic performance. We also discuss the reaction mechanism of CO_2_ methanation and find that there are roughly three pathways, namely the CO pathway, the formate pathway, and the direct hydrogenation of CO_2_ to CH_4_. Based on this discussion, we suggest that the resistance to carbon accumulation and sulfur poisoning should be taken into account when improving the performance of the catalyst. Moreover, it is necessary to try more new materials and preparation methods, consider the effect of support morphology on catalyst performance, and use bimetallic catalysts with better performance.

## Figures and Tables

**Table 1:** Basic steps of CO_2_ methanation proposed via the CO formation pathway (left) and the formate formation pathway (right) (s refers to surface sites) [42]. Copyright (2021) Catalysis Today.

CO Formation Path Way	Formate Formation Path Way
$H_{2},g+2s=2H_{-s}$ (1a)	$H_{2},g+2s=2H_{-s}$ (2a)
$CO_{2},g+2s=CO_{-s}+O_{-s}$ (1b)	$CO_{2},g+s=CO_{2-s}$ (2b)
$CO_{-s}=CO,g+s$ (1c)	$CO_{2-s}+H_{-s}=HCOO_{-s}+s$ (2c)
$CO_{-s}+s=C_{-s}+O_{-s}$ (1d)	$HCOO_{-s}+H_{-s}=HCO_{-s}+OH_{-s}$ (2d)
$C_{-s}+4H_{-s}=CH_{4-s}+4s$ (1e)	$HCO_{-s}+H_{-s}=CH_{-s}+OH_{-s}$ (2e)
$CH_{4-s}=CH_{4},g+4s$ (1f)	$CH_{-s}+3H_{-s}=CH_{4},g+4s$ (2f)
$O_{-s}+2H_{-s}=H_{2}O+2s$ (1g)	$OH_{-s}+H_{-s}=H_{2}O,g+2s$ (2g)
